# Road traffic and landscape characteristics predict the occurrence of native halophytes on roadside verges

**DOI:** 10.1038/s41598-022-05084-3

**Published:** 2022-01-25

**Authors:** R. Fekete, H. Bak, O. Vincze, K. Süveges, V. A. Molnár

**Affiliations:** 1 Department of Botany, University of Debrecen, 4032 Debrecen, Hungary; 2 Department of Tisza Research, Institute of Aquatic Ecology, Centre for Ecological Research, 4026 Debrecen, Hungary; 3 Evolutionary Ecology Group, Hungarian Department of Biology and Ecology, Babeș-Bolyai University, 400006 Cluj-Napoca, Romania

**Keywords:** Ecology, Urban ecology

## Abstract

Road management practices, such as winter de-icing create ideal habitats and competitive advantage for salt-tolerant species. We aimed to map the occurrences of halophytes along roads in Hungary. Furthermore, we tested factors that might play a role in the roadside occurrences of five chosen native halophytes from rare to common, we encountered during our field surveys. These were *Festuca pseudovina*, *Limonium gmelinii* subsp. *hungaricum*, *Podospermum canum*, *Puccinellia distans* and *Spergularia media*. We found, that at least one halophyte species was documented in 71% of the total sampling points. Germination experiments indicated that substrate salt concentration significantly decreased germination rates in each of the five species, but in case of *L. gmelinii* subsp. *hungaricum*, or *P. distans* germination occurred on extremely high salt concentrations. Traffic intensity, the presence of other halophytes at the sampling point and the presence of a given species in the surrounding landscape had a significant positive effect on the occurrence of four of the five model species. Our results suggest that the studied species are mostly in the early stage of their roadside spread, colonizing roadsides close to their native distribution ranges. The possibility of a future range expansion along roads cannot be excluded.

## Introduction

In recent decades, huge road network developments have taken place across the globe, creating sophisticated, international road network axes. The paved road network in the European Union by 2017 is considered to be one of the most extensive per unit area, with a total length of almost five million km^1^. In 2019, the total length of paved roads in Hungary was 32,204 km, of which the main network was 9077 km, while the secondary network was 23,127 km^2^. The explosive growth of global road network has a negative impact on the occurrence and diversity of most groups of organisms^3^. These negative impacts are direct consequences of the habitat fragmentation, disturbance, pollution, direct damage to individuals or the spread of invasive species associated with roads and traffic^4,5^. Besides expanding road networks, the worldwide growth of trade and transportation facilitates the spread of plants associated with human mobility^6,7^. For instance, mud adhering to vehicles can contain large amounts of propagules^8,9^. An experimental study demonstrating that more than 100 plant species germinated from mud adhered to a single vehicle that traveled 15,000 km^10^. Moreover, the airflow generated by vehicles also facilitates the spread of species characterized with low seed mass^11^. Furthermore, Vitalos & Karrer^12^ drew attention to the role of roadside mowing vehicles in seed dispersal.

Roads are often regarded as ecological corridors because they play an important role in the spread of plants^13^. The spread of alien and invasive species along roads has long been known and has been reported in several studies^14–19^. As early as the 1980s, dispersal was observed in insect and plant species along expressway networks and railroads^20,21^. By the twenty-first century, as a result of the development of international road networks, the spread across countries has also accelerated, when cross-border and long-distance spread has increased in frequency, also resulting in an increased risk of dispersal of invasive species^22^. Invasive and other alien species may also be introduced with soils used in road constructions, that are transported from remote sites and contain plant propagules^23^. Additionally, road management practices, aiming to create safe conditions for traffic alter the environment in the vicinity of roads. For instance, the use of salt for winter de-icing. NaCl has been used for de-icing in Hungary since the 1960s^24^, but it has several negative effects on the environment and vegetation. It pollutes surface waters, drinking and groundwater, and also promotes corrosion of vehicles and bridges^25^. Increased soil salt content alters pH, nutrient availability, and causes osmotic stress, thereby altering vegetation composition and promoting the spread of stress-tolerant, halophytes^26^. Furthermore, it causes leaf damage to some tree species and have negative impacts on several arthropod communities^27^ and environmental impacts can be recorded 30 m away from the road by water run-off^28^. De-icing salts have negative impact on freshwater and wetland flora, fauna and biogeochemistry^29^. Very large amounts of salt are deposited in the soil close to the roads, but the concentration decreases rapidly with the distance from the road’s edge^30^. Halophytes are species that can survive and reproduce on soils with salt concentration above 200 mM, and they represent only about 1% of the world’s flora^31^. Facultative halophytes are able to survive without salt, but low levels of soil salt concentration help their growth, while obligate halophytes are only able to survive and show optimal growth under saline conditions^32–35^. However, the latter are probably species restricted to extreme environments due to their low competitive ability and greater salt tolerance^32^. Halophytes are now widely known to be able to spread along roads and colonize inland areas across Europe^36–39^ The first alien, coastal halophyte found in the Hungarian road network was *Plantago coronopus*, first documented in 2013 along the M1 and M70 motorways^40^ and has become widespread along Hungarian roads^41–43^. In 2016, another coastal, salt-tolerant species was found in Hungary, namely *Cochlearia danica*^44^. Native halophytes can also be found along roads in Hungary occasionally surprisingly far from their native distribution range^45^. The roadside spread of plant species could be a very serious problem in conservation, since the spread of non-native species from roadsides into natural habitats has become a well-known phenomenon in recent years^46–48^. This is also known in the case of a coastal halophyte, *P. coronopus,* that is spreading successfully along roads, and have already become an invasive alien species in the USA, where it threatens endangered plant species in coastal habitats^49,50^. However, in some of its natural habitats *P. coronopus* is considered as an endangered species^51^. Thus, it is important to follow roadside occurrences of plants and to investigate which environmental factors or species characteristics are responsible for their rapid spread. Furthermore, halophytes represent an interesting group due to their salt tolerance, that facilitates their endurance to roadside circumstances.

Here we conducted a comprehensive field survey of halophytes along Hungarian roads. Based on this field survey we chose five native, characteristic halophyte species as model species in this study. Our aim was to determine which environmental or road characteristic influence the differences in roadside occurrences of these five halophytes found in different frequencies along roads. These species were *Festuca pseudovina* Hack. ex Wiesb*.* (Poaceae)*, Limonium gmelinii* (Willd.) Kuntze subsp. *hungaricum* (Klokov) Soó (Plumbaginaceae)*, Podospermum canum* C.A.Mey. (Asteraceae)*, Puccinellia distans* (Jacq.) Parl. (Poaceae) and *Spergularia media* (L.) C.Presl (Caryophyllaceae).

In this study we specifically aimed to 1) survey the roadside occurrences and distribution of the mentioned five halophytes along Hungarian roads, as well as to document the microhabitats occupied by these (e.g. distance to the road edge); 2) test their potential seed production, as an important factor in dispersal and to explore the effect of substrate NaCl content on the germinability of their seeds; 3) explore whether the following four environmental factors predict their roadside occurrences and frequencies: altitude, the number of other halophytes (besides the model species) present at the sampling points; traffic intensity; and finally the presence of native population of the candidate species in the surrounding landscape. These analyses will help us reveal whether roadsides function as ecological corridors for these species or the occurrences along roads is likely to be determined by the presence of the species in natural populations in close proximity to the sampled road sections.

## Materials and methods

### Road surveys

In order to survey roadside occurrences of the five model species we carried out fieldwork between April and September 2018. We surveyed a total of 519 random sampling points along roads, covering an approximately 2600 km long road section across Hungary, with sampling points randomly designated every 4–5 km, at points where the traffic situation and rules allowed stopping. At every sampling point, we surveyed the roadside along a 100-m-long transect and we recorded the presence or absence of the five halophyte model species and we also recorded the presence of other halophytes besides our model species. The number of individuals belonging to these species, their distance from the edge of the road along with geocoordinates and altitude of the sampling locations were also recorded. The distance of individuals from the road edge was examined at a width of 6 m from the road. Given that cars cannot stop at random points on motorways, these surveys were restricted to gas stations and rest areas. We used the online database of Hungarian Public Road Nonprofit Pte Ltd Co.^2^ to categorize the surveyed roads as follows: motorways, main roads (1st main roads, 2nd main roads) and lower category roads [i.e. connecting roads (connecting settlements), conjucting roads (connecting settlements to the national road network), roads leading to railway station, unknown numbered roads].

### Road and landscape parameters

To characterize roads, we gathered information about the cross-sectional annual average daily traffic data of road sections surveyed during this study. The latter data was obtained for the year 2018 from the online database of the Hungarian Public Road Nonprofit Pte Ltd Co.^2^. In order to assess whether nearby occurrence of a model species affected its roadside occurrences, we gathered information on the number of occurrences of the studied species in flora mapping quadrats (6,25 × 5,55 km) surrounding our surveyed roadside sampling points using the Hungarian Flora Mapping Database^52^. In the latter case we calculated the total number of occurrences of a species in the quadrate in which the sampling point was located as well as in the eight quadrates surrounding this using Quantum GIS version 3.4^53^. We gathered this data for all five model species.

### Reproductive traits and germination experiments

**Figure 1:**
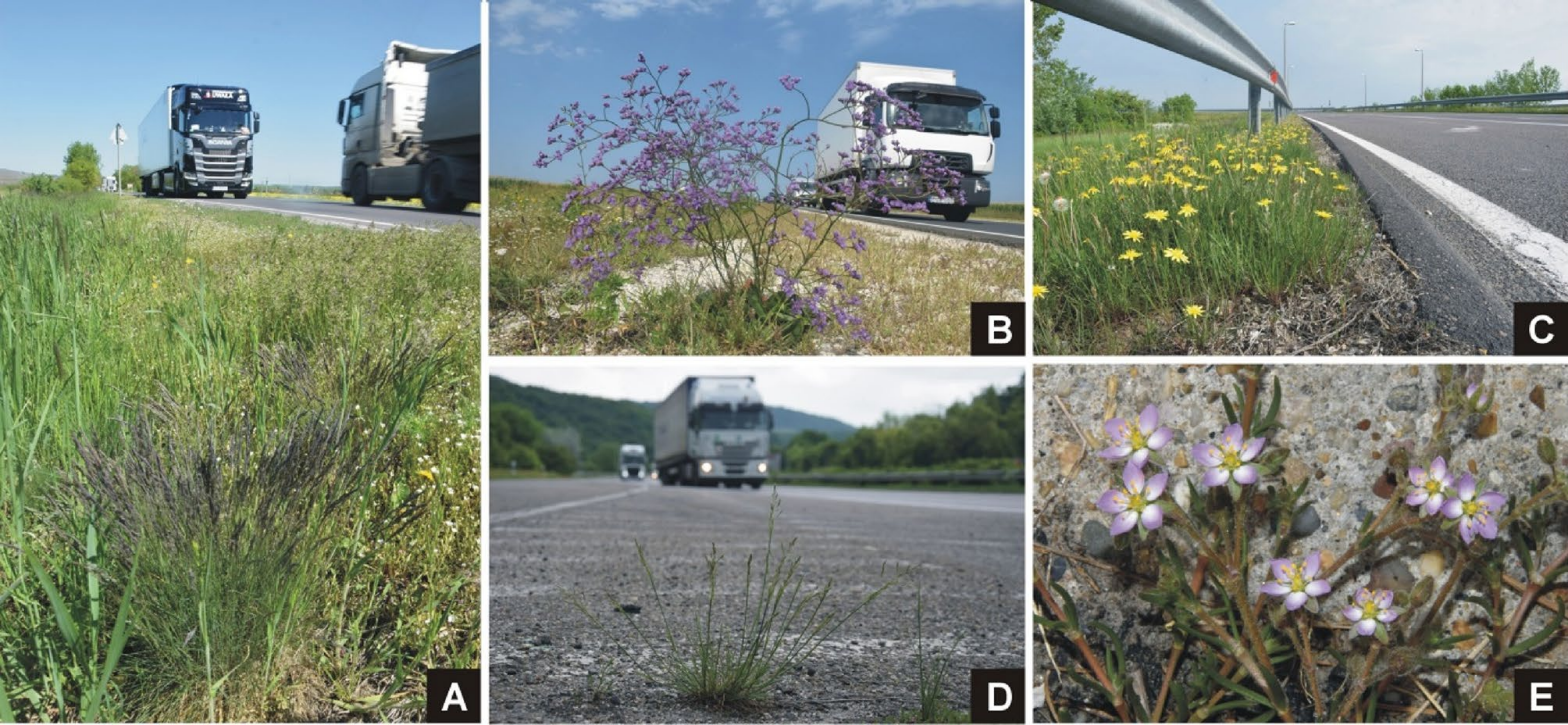
The five model species surveyed. A *Festuca pseudovina,* B *Limonium gmelinii,* C *Podospermum canum,* D *Puccinellia distans,* E *Spergularia media*. Photographed by A. Molnár V.

For species identification we followed the *New Hungarian Herbal. The Vascular Plants of Hungary*^54^*.* All five species studied have highly characteristic morphology and their identification is straightforward. No voucher specimens were collected, but occurrences were documented using photographic evidence (Fig. 1).

In order to estimate individual seed production by our model species, we collected 30 individuals/species during fieldwork in 2018. We determined their following characteristics to assess seed production: number of flowering shoots of 30 individuals, number of inflorescences per shoot (in this case also the number of flowers per inflorescence in 30 inflorescences) or number of flowers per shoot, and the number of seeds per fruit in 30 fruits. Thousand seed mass of the species was determined following Török et al. 80, by calculated it from the data obtained by measuring the mass of 3 × 100 seeds.

The germinability of the seeds of the five species in different salt concentrations was tested in October 2019 in an in vitro experiment. Seeds did not need stratification. Seeds for the germination test of *Limonium* were collected on 16 September 2019 near Balmazújváros, seeds of *Podospermum* were collected on 02 June 2019 near Karcag, seeds of *Spergularia* and *Festuca* were collected on 25 June 2019 near Sárszentágota, while *Puccinellia* seeds were collected from the side of the road 33 between Debrecen and Hortobágy on 18 June 2019. Prior to the start of the experiment all seeds were stored at room temperature (22–23C°) under similar conditions. Seeds of *Festuca*, *Podospermum*, *Puccinellia* and *Spergularia* were stored for approximately four month and seeds of *Limonium* were stored for one month. Seeds were placed to germinate in Petri dishes containing 1% agar substrate with one of the following 14 different concentrations of NaCl in mass percent [m/m%] (and molar solutions (mM) and soil salinity of saturated extract (ECe in dS/m) converted following Food and Agriculture Organization (FAO) classification^55^: 0% (control); 0.15% (25.67 mM, 2.57dS/m); 0.30% (51.33 mM, 5.13dS/m); 0.45% (77.00 mM, 7.70 dS/m); 0.60% (102.67 mM, 10.27dS/m); 0.75% (128.34 mM, 12.83dS/m); 0.90% (154.00 mM, 15.40dS/m); 1.05% (179.67 mM, 17.97dS/m); 1.20% (205.34 mM, 20.53dS/m); 1.35% (231.01 mM, 23.10dS/m); 1.50% (256.67 mM, 25.67dS/m); 2.00% (342.23 mM, 34.22 dS/m); 2.50% (427.79 mM, 42.78dS/m); 3.00% (513.35 mM, 51.34dS/m). A total of 2100 seeds were placed to germinate per species, 50 seeds per Petri dish, and each salt concentrations was tested in triplicate. Germinability test was started on 13 October 2019 performed in parallel for the five species and lasted for 33 days. Petri dishes were kept at room temperature, close to a window, subject to natural light conditions throughout the experiment. The number of germinated seeds was checked and recorded daily. Criteria for germination was the appearance of the first green parts, the cotyledon (in case of *Limonium*, *Podospermum* and *Spergularia*) or coleoptile (in case of *Festuca* and *Puccinellia*).

### Data analyses

Statistical analyses were carried out in R (version 3.4.1)^56^. In order to test whether there is a difference between species regarding their distance from the edge of the road (thus testing the occurrence in different microhabitats) we built linear mixed effect models (LMMs) using package lme4^56,57^. We used the distance from the road of individual plants as a dependent variable, while species identity was included as sole explanatory variable. Given that observations within a sampling point are non-independent, sampling point identifier was included as a random factor in the model. To assess significance, we used the Satterthwaite approximation for degrees of freedom as recommended to minimize type I error (1) and implemented in the lmerTest package (2) and likelihood ratio tests for multiple-df tests.

To analyze germinability of seeds at various salt concentrations we used a binomial GLMM using package lme4^57^, where germination outcome (germinated/non-germinated) of seeds was used as dependent variable and NaCl concentration was the sole explanatory variable, included as a second-degree orthogonal polynomial. All models included the identifier of the Petri dish as a random factor. If the non-linear effect was not significant, we re-fitted the model using only the linear NaCl concentration as an explanatory variable. Predicted germination probabilities and associated prediction intervals were obtained using the ’predictInterval’ function^58^.

In order to explore whether landscape flora and road characteristics predict roadside occurrences of the five species studied here, we used generalized linear models (GLM) using package lme4^57^, with binomial error distributions (i.e. for presence/absence data) built separately for each of the five model species. Presence or absence of a given model species was used as dependent variable and we used altitude, traffic intensity, number of other halophyte species (besides our five model species) found at the sampling point, and the sum of occurrences of the candidate species in the surrounding landscape (flora mapping quadrats) as explanatory variables in the models. Only ten species were considered as ‘other halophytes’ in the analysis, namely species whose phenology allowed detection during the period of our field surveys. These were the following: *Artemisia santonicum, Atriplex tatarica, Festuca pseudovina, Hordeum hystrix, Limonium gmelinii* subsp*. hungaricum, Podospermum canum, Puccinellia distans, Spergularia maritima, S. rubra, S. salina*. In a GLM where we used a given model species as dependent variable, this species was not included in the sum of other halophytes. We further included the county as random factor.

Nomenclature used in this work follows The Plant List (2013)^59^.

### Ethical statement

The presented experimental research and field studies on plants, including the collection of plant material, comply with relevant institutional, national, and international guidelines and legislations. Plant material was collected from public lands and none of the studied species are protected by law in Hungary, therefore collection did not require special permits.

## Results

### Mapping halophytes along roads in Hungary

**Figure 2:**
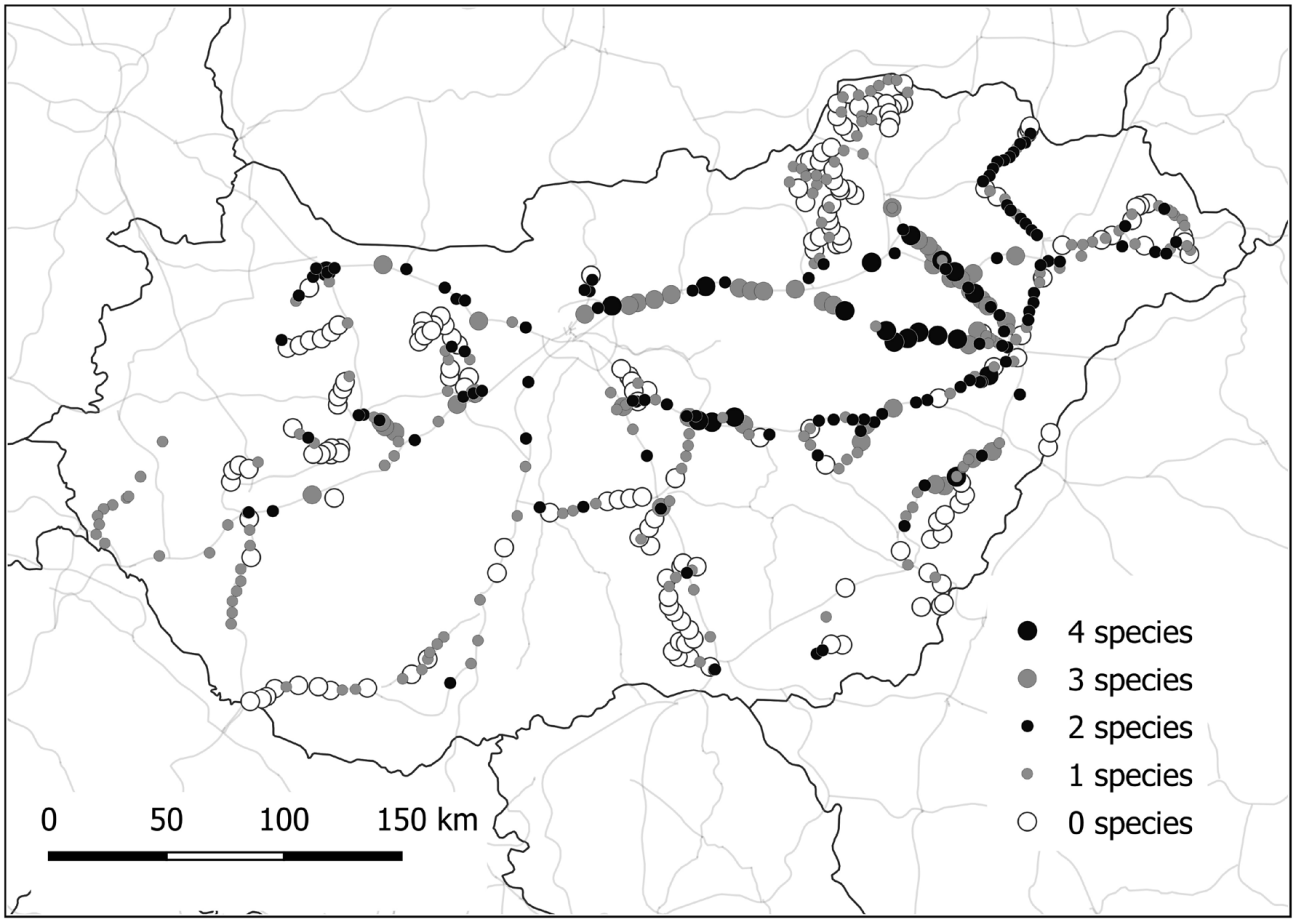
Map showing the distribution of sampling points and the number of species encountered along Hungarian roads.

Here we surveyed a total of 517 random sampling points at roadsides in Hungary, covering different road categories with various traffic and disturbance intensities (Fig. 2). The survey resulted in the documentation of 15 halophyte species besides our model species occurring in roadside verges of public roads (Table S1). The large majority (over 71%) of the surveyed sampling localities harboured at least one halophyte species. Only 27 points with salt-tolerant plants harboured none of the five model species studied in detail here.

The most commonly documented halophyte along roads in Hungary was *Puccinellia distans*, whose presence was documented at more than half of our sampling points, with a total of nearly 19,000 individuals. The rarest of the five model species was *Spergularia media*, which was found in only six sampling locations (Table 1).

**Table 1:** Summary of the documented roadside occurrences of the five target species. Prevalence, total and average number of individuals across all sampling points is presented for each species.

Species	Percentage of sampling points where species was present	Total number of individuals documented	Average number of individuals at sampling points
*Festuca pseudovina*	36.56	11,074	21
*Limonium gmelinii* subsp. *hungaricum*	9.86	575	1
*Podospermum canum*	19.92	5377	10
*Puccinellia distans*	51.45	18,952	37
*Spergularia media*	1.16	182	0.4

Interestingly, when the relative frequency of the five target species was assessed for different road categories, *Festuca, Podospermum* and *Puccinellia* showed similar frequency along motorways. All three of these species were present at more than the 60% of the sampling points taken along motorways. *Limonium* was less frequent along motorways, while *Puccinellia* was the most frequent in this category (Table 2).

**Table 2:** Ratio of presence of the five species in the sampling points at different road categories [number of points with the presence of the species/ number of sampling points (ratio)].

Species	Motorways	Main roads	Lower category roads
*Festuca pseudovina*	47/76 (61.84%)	120/247 (48.58%)	22/194 (11.34%)
*Limonium gmelinii* subsp*. hungaricum*	5/76 (6.57%)	39/247 (15.79%)	7/194 (3.61%)
*Podospermum canum*	47/76 (61.84%)	44/247 (17.81%)	12/194 (6.19%)
*Puccinellia distans*	48/76 (63.16%)	158/247 (63.97%)	60/194 (30.93%)
*Spergularia media*	6/76 (7.89%)	0/247 (0%)	0/194 (0%)

Statistical analysis indicated that individuals belonging to the five halophyte species were situated at different distances from road (LMM, p < 0.001|). Individuals of *Spergularia* (41.5 ± 86 cm) occurred closest to the road edge, followed by *Puccinellia* (78.35 ± 56.25 cm), *Podospermum* (84.18 ± 73 cm), *Limonium* (97.95 ± 64.58 cm) and generally *Festuca* (192.6 ± 86.5 cm) individuals were found the farthest among the five species.

### Reproductive traits and germination experiment

We have found that the estimated potential seed production per individual was the highest in case of *Limonium,* but was similarly high in the case of *Spergularia* (Table 3)*.*

**Table 3:** Estimated reproductive traits of the five model species.

Species	Potential seed production (average seed number/individual)	Thousand seed mass (g) (Török et al. 81, 82)
*Festuca pseudovina*	453	0.2633
*Limonium gmelinii* subsp*. hungaricum*	2288	0.6736
*Podospermum canum*	282	4.105
*Puccinellia distans*	297	0.1443
*Spergularia media*	2148	0.3327

The germination experiment showed marked germinability differences among the five model species, with a high proportion of seeds germinating in the case of *Festuca*, but only a few germs being observed in the case of *Spergularia* (Table 4). Some seeds germinated already by the first day of the experiment in case of each species, but this usually happened only at lower salt concentrations (0.00–0.30%). At the highest salt concentration (3.00%) only *Limonium* was able to germinate in very small numbers. All species germinated at the four lowest concentrations (0.00%; 0.15%; 0.30%; 0.45%) and all species had the highest germination rate on the control media and the lowest germination rates occurred on the highest NaCl concentrations.

**Table 4:** Summary of the germination experiment. Percentage of germinated seeds from the 2100 tested seeds/species, the highest NaCl concentration where germination occurred and germination percentages at the highest concentration where germination occurred is shown.

Species	Percentage of germinated seeds (%)	Highest concentration (m/m% NaCl)	Percentage of germinated seeds on the highest concentration (%)
*Festuca pseudovina*	34.43	1.50	7.33
*Limonium gmelinii* subsp. *hungaricum*	11.66	3.00	0.67
*Podospermum canum*	13.43	0.90	0.67
*Puccinellia distans*	4.67	2.00	1.33
*Spergularia media*	1.95	1.05	0.67

**Figure 3:**
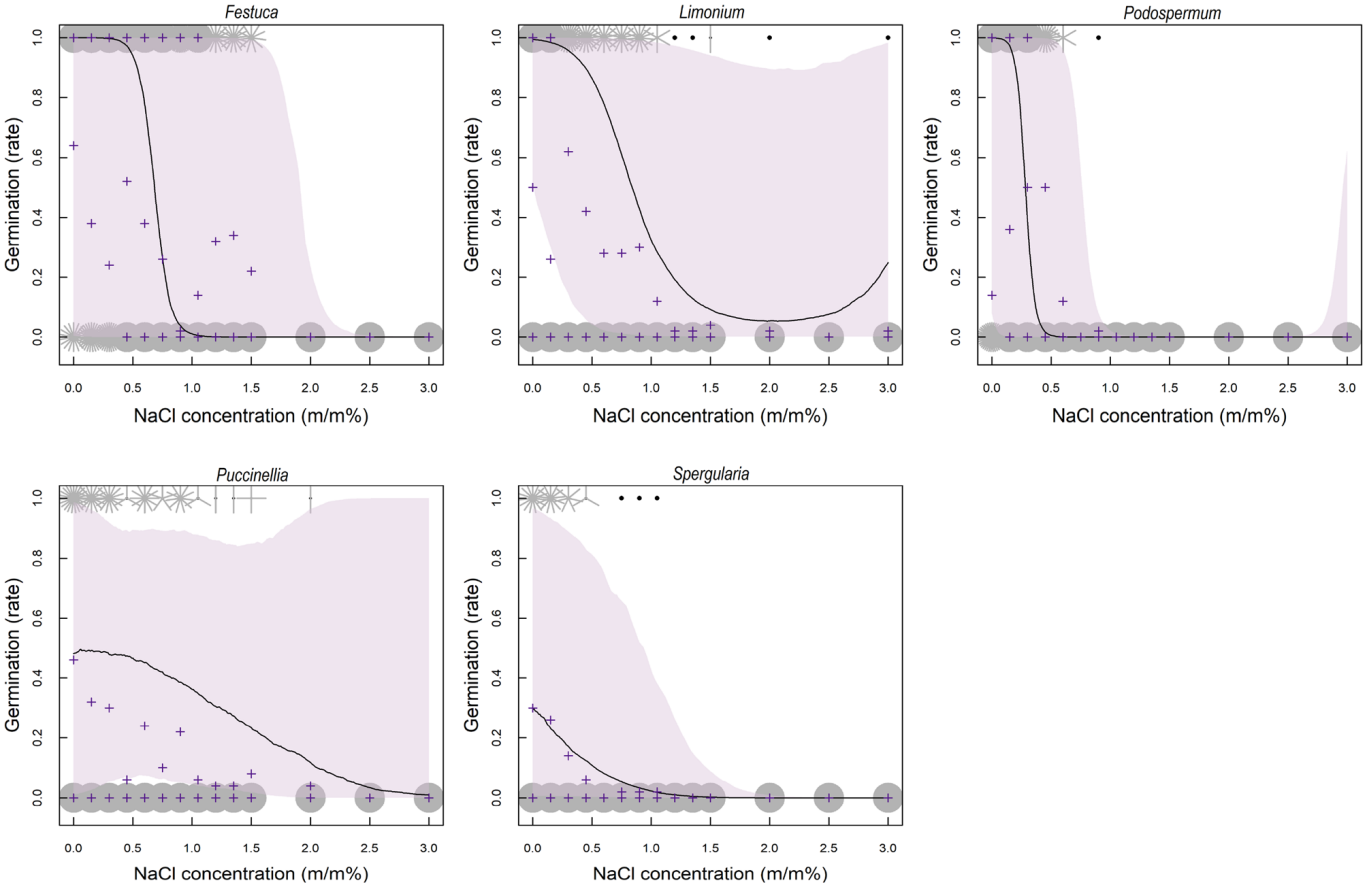
Sunflowerplots showing the association between germination and substrate NaCl concentration in the five model species. Petals represent individual seeds, black dots mark single data point, purple pluses mark average germination rate per petri dish. Predictions and associated confidence intervals were obtained from respective GLMM models.

Germinability decreased significantly or marginally significantly (in case of *Puccinellia*) with increasing substrate NaCl concentration as shown by GLMMs performed separately for each species (Table 5). The effect of NaCl concentration was non-linear in case of *Limonium* and *Podospermum*, but was linear in case of *Festuca, Puccinellia* and *Spergularia* (Fig. 3).

**Table 5:** Effect of NaCl concentration on germination: (poly (NaCl concentration) 1 shows the linear effect and poly (NaCl concentration) 2 shows the non-linear effect of NaCl on germination (germinated/non-germinated seeds)).

	Estimate	SE	t-value	p-value
*Festuca pseudovina*				
Intercept	− 5.036	1.518	− 3.318	0.00091
NaCl concentration (linear term)	− 495.16	25.706	− 19.262	< 0.001
*Limonium gmelinii* subsp. *hungaricum*				
Intercept	− 7.038	1.548	− 4.547	< 0.001
NaCl concentration (linear term)	− 93.241	20.42	− 4.566	< 0.001
NaCl concentration (non-linear term)	66.956	18.739	3.573	< 0.001
*Podospermum canum*				
Intercept	− 11.833	2.094	− 5.65	< 0.001
NaCl concentration (linear term)	− 292.15	39.916	− 7.319	< 0.001
NaCl concentration (non-linear term)	280.5	22.322	12.566	< 0.001
*Puccinellia distans*				
Intercept)	− 7.459	1.921	− 3.882	< 0.001
NaCl concentration (linear term)	− 49.722	28.523	− 1.743	0.08129
*Spergularia media*				
Intercept	− 9.452	1.805	− 5.238	< 0.001
NaCl concentration (linear term)	− 186.27	37.518	− 4.965	< 0.001

### Effect of road and landscape characteristics on roadside occurrences

Traffic intensity had a significant positive effect on the occurrence of *Festuca, Podospermum Puccinellia* and *Spergularia*, but had no effect on *Limonium*. The probability of occurrence at roadsides increased significantly with increasing number of other halophytes present at the sampling site in all species except *Spergularia*. Moreover, sum of occurrences in the surrounding flora mapping quadrats significantly positively influenced occurrence probability in *Festuca*, *Limonium, Podospermum* and *Spergularia*, but not in *Puccinellia* (Table 6). Altitude had significant negative effect on the occurrence of only two halophytes, *Festuca* and *Limonium*.

**Table 6:** Results of GLMMs exploring the link between environmental factors (altitude of the sampling point, annual average daily traffic data of the road section and roadside occurrences (presence/absence) of the five model species (only minimal models are shown) including the 517 sampling points.

	Estimate	SE	t value	p value
*Festuca pseudovina*				
Intercept	− 1.19	0.26	− 4.59	< 0.001
Altitude	− 0.87	0.37	− 2.36	0.018
Number of other halophytes	1.24	0.16	7.82	< 0.001
Traffic intensity	0.65	0.13	4.88	< 0.001
Sum of occurrences in the flora mapping quadrats	0.47	0.19	2.52	0.012
*Limonium gmelinii* subsp. *hungaricum*				
Intercept	− 5.15	0.75	− 6.86	< 0.001
Altitude	− 4.69	1.18	− 3.98	< 0.001
Number of other halophytes	0.75	0.19	3.98	< 0.001
Sum of occurrences in the flora mapping quadrats	0.60	0.18	3.24	0.001
*Podospermum canum*				
Intercept	− 2.15	0.36	− 5.91	< 0.001
Number of other halophytes	0.78	0.16	4.76	< 0.001
Traffic intensity	0.78	0.15	5.08	< 0.001
Sum of occurrences in the flora mapping quadrats	1.01	0.22	4.64	< 0.001
*Pucinellia distans*				
Intercept	− 0.07	0.33	− 0.22	0.83
Number of other halophytes	0.58	0.14	3.99	< 0.001
Traffic intensity	0.30	0.14	2.15	0.032
*Spergularia media*				
Intercept	− 6.09	1.05	− 5.80	< 0.001
Traffic intensity	0.98	0.27	3.61	< 0.001
Sum of occurrences in the flora mapping quadrats	0.73	0.24	3.08	0.002

## Discussion

Halophytes are increasingly recognized to colonize disturbed roadside verges. In this study we highlight the frequency and species richness of halophytes colonizing roadside verges, by showing that over 70% of random sampling points harbour at least one of the 20 identified species colonizing verges. Moreover, by examining the occurrences of five commonly encountered halophyte species in Hungary we highlight the role of traffic intensity, local conditions (e.g. presence of other halophytes at sampling) as well as the influence of the surrounding landscape’s flora in influencing the probability of roadside colonization in several halophytes.

### Roadside occurrences

The surveyed model species all had been documented along roads before. Previously, *Festuca pseudovina* had only one documented roadside occurrence in Hungary^60^ and one in Austria [referred to as *Festuca valesiaca* subsp*. parviflora*]^61^. More frequent occurrence of *Limonium gmelinii* subsp. *hungaricum* has been documented along Hungarian roads^45,62–64^, as well as in other European countries. It was found along roads in the Czech Republic next to motorways^64^, then in Austria^65^ and later in Germany^66^. In all three cases, *L. gmelinii* was registered as a new, adventive species in the flora of the country. *Podospermum canum* has also been registered to occur along public roads in Hungary^40,44,45^. In addition, it was recorded at roadsides in Austria^64,65^ as *Scorzonera cana*, Greece [referred to as *Scorzonera cana*^69^], Turkey [referred to as *Scorzonera cana*^70^] and Lebanon^71^. One of the most commonly encountered halophytes studied here is the *Puccinellia distans*, representing the first halophyte to be documented along roads in Hungary^72^. This species is considered as one of the most common halophytes along European roads, appearing on roadsides in many countries, including Great Britain^73,78^, Germany^74,75^, Poland^76^, the Czech Republic^37^, the Netherlands^77^ or France^78^. The species has been found along North American roads as well^79,80^. Finally, the occurrence of *Spergularia media* along roads is well documented in Hungary [39, 2018] as well as in several other countries, such as Austria^81^, the Czech Republic^82^, Germany^68^, the United Kingdom^73^, Ireland^83^ or in the USA^84^.

Data generated by our field surveys indicate that roadsides provide potentially suitable habitats for halophytes. It is likely that due to the increased salinity of the soil in the proximity of roads, halophytes find favorable conditions here, where their salt tolerance might provide them with competitive advantage over other species. *Limonium* did not form large monodominant patches along roadsides, whereas *Podospermum*, *Festuca*, and *Puccinellia* occurred in many cases in dense contiguous patches along roadsides. Several other disturbance-tolerant species occurred at our surveyed points, the most common of which were the *Matricaria recutita* and *Atriplex tatarica*.

The spread of halophytes was hypothesized to be more likely along roads with higher-traffic intensity (e.g. motorways) due to higher air turbulence and due to the passing of more vehicles potentially transporting seeds. In accordance with this hypothesis, we found that three of our five model species occurred most frequently along motorways, however*, Limonium* and *Puccinellia* were more common along roads of intermediate traffic intensity. Based on our results and our personal observations in the field we believe that the spread of most halophytes in Hungary began along lower-category roads that pass through the natural habitats of the focal species, where roadside verges form a natural transition to the surrounding landscape. As a second step, propagules from established populations along lower category roads reach motorways where they can start a faster spread due to a more intense traffic. In support of this hypothesis, a series of halophytes documented on road edges during our field survey were found only along main roads, but not along motorways. These inlcuded *Hordeum hystrix*, *Plantago tenuiflora, P. maritima, Lepidium perfoliatum, Myosurus minimus, Artemisia santonicum, A. ponticum* and *Spergularia salina*. It remains to follow the spread of these species along roads, especially from already established roadside populations. *Plantago maritima* was also found along roads in Scotland^85^. However, during our roadside surveys, *Hordeum hystrix* was found only at a few points nearby original saline habitats, propagules of this species was found to be carried by cars in large numbers in Australia^86^.

A proposed key component determining the distance of halophyte individuals from the road is the distribution of de-icing salt content within the verge, as well as the diminution of the competitive advantage with increased distance from the road’s edge of halophytes over other ruderal, stress-tolerant species. In case of our five model species, each appeared to occupy different microhabitats at the roadsides, which might also reflect their competitive ability. *Festuca* occurred generally farthest from the road edge mostly in dense closed grassy places and *Spergularia* individuals were the closest to the road edge, but mostly where free surfaces were available. These microhabitats are very similar to the natural microhabitats they colonize on the salt steppes regarding the relative vegetation cover and bare ground^87^.

#### Reproductive traits and germination experiments

Previous studies have shown that low seed weight is one of the most important functional traits in plant dispersal, as low seed weight is usually associated with significant seed production, persistent seed bank, and efficient wind propagation^88,89^. Upon comparing to the average thousand-seed weight of Hungarian salt-tolerant species, we found that *Puccinellia, Festuca* and *Spergularia* had much lower seed weight, than the overall average. We gathered the seed mass of 60 native salt steppe species based on Török et al.^90,91^ to compare them with the seed mass of our model species. The average seed mass of them was 0.7661 g () and comparing to this, *Podospermum* was the only species with higher seed mass while *Limonium* was close to the average value. The high seed mass of the latter two species can be compensated for by other reproductive traits in terms of propagation. In case of *Limonium* the higher seed mass is accompanied by significant individual seed production, while the propagation of large seeds of *Podospermum* is winged by the pappus.

Previous studies have shown a positive correlation between seed number per fruit and spatial spread^92^. Here we found that the two rarest of our model species (*Limonium* and *Spergularia*) have the largest seed productions, while the most widespread model species, *Pucinellia* produced the second lowest seed counts, indicating that seed production is unlikely to be the key factor explaining variation in the distribution of native saline species along roads. Another important trait could be the life-form of these halophytes, most of them being hemicryptophytes, which are well known to be high resistant to mowing thanks to their overwintering organs. The success of our model species along the roads can also be increased by their high degree of salt tolerance, which was also demonstrated by our germination experiment. In consistent with our experiment, other studies have also been documented the negative effect of NaCl on germination rates in case of *Spergularia media*^93^ and *Puccinellia distans*^94^. Soils with a soluble salt content of 0.25–0.50% can already be classified as highly saline, where only few halophytes can survive^95^. In all five of our model species, germination was documented at concentrations much higher than this threshold. Although NaCl alone does not represent the composition of the soluble salt-content of saline soils^95^. According to the FAO classification^55^, four of our model species germinated on NaCl concentrations that corresponded to very strongly saline soils in nature. On such high salt concentrations only a few tolerant crops are able to produce satisfactory yields. Moreover, *Podospermum* germinated on substrates that corresponded in salt concentration to strongly saline circumstances. Halophytes requires a series of physiological and morphological adaptations in order to make them truly tolerant to high salt concentrations. The regulation of increasing salt levels in plant tissue has led to the induction of specific changes in plant cell tissue and organs through structural and physiological alterations^96^. One example is the presence of basal leaves, as in the case of *Limonium* and *Podospermum*, which may serve as a strategy to avoid salts to penetrate into upper plant organs^97^. Leaf rolling is a xeromorphic strategy, but have a great role in aiding the effects of physiological drought caused by salinity in *Festuca* and *Puccinellia*. Dicotyledonous halophytes (like *Limonium gmelinii* subsp. *hungaricum* or *Spergularia media*) show xeromorphic characteristics, such as thick succulent leaves, that facilitates sufficient water supply^96,98^. *Limonium,* furhtermore has salt-glands facilitating the recretion of salts^99^.

Generally, monocotyledonous halophytes, that show optimal growth under 50 mM NaCl^100^ are characterized by lower salt tolerance than dicotyledonous halophytes, that shows optimal growth up to 250 mM NaCl^101^. According to Pătruţ et al.^102^ *Limonium* and *Puccinellia* belong to species adapted to intensely salinized biotopes, while *Festuca* and *Podospermum* adapted to soils with lower, but different degrees of salinization. This also reflects our results, since *Limonium* and *Puccinellia* were characterized by the highest salt tolerance according to the germination experiment. Overall we conclude, that the salt tolerance of our model species does not predict their frequency along roads, suggesting that extreme salt tolerance might not be an important factor in the roadside success of halophytes.

#### Factors influencing roadside occurrences

The roadside occurrences of our studied species seemed to be strongly positively influenced by traffic intensity and by the distribution of the focal species in the surrounding landscape. Furthermore, the number of other halophytes present at the sampling points was also a significant predictor of higher probability of presence. For two species, *F. pseudovina* and *L. gmelinii* subsp. *hungaricum*, a significant negative effect of altitude was observed. Our result indicating the positive effect of traffic intensity is not surprising, since several studies have already indicated the role of traffic in the spread of plant species^37,103^. The correlation between the number of other halophytes and the presence at the roadside of our model species indicates that it is likely that salt-tolerant species colonize these edges from nearby saline habitats, potentially from habitats identical to those where our model species originates. On the other hand, more halophytes at sampling locations might simply be indicative of local conditions that are highly suitable for salt-tolerant taxa, and therefore for our model species. In four of our model species the positive effect on roadside occurrence of the presence in the surrounding flora mapping quadrats clearly indicates that they currently mostly occur at roadsides where they also occur in the surrounding landscape. In the case of our most common roadside model species, *Puccinellia*, this correlation could not be detected. This probably indicates the fact that this species has already reached the stage of faster roadside dispersal outside its native distribution range. It is also important to note, that *Puccinellia* is known from 521 quadrats from Hungarian Flora Mapping Database, whereas our rarest roadside model species *Spergularia* is known only from 34 quadrats, which shows that their frequency along roads is also strongly influenced by frequency in the landscape.

In conclusion, several of our native salt-tolerant species appear to be at a stage of local colonization of roadsides, mostly situated within their native range. However, Austrian, Czech and German roadside occurrences of *Limonium*^61–63^ may be good examples of the potential long-term spread of our native halophytes, which seems to be an occasional phenomenon for now, but with increasing traffic intensity it is likely to occur more frequently. These results highlight how certain species (even potentially invasive ones) reach the roadside and exit their original range. This provides data also for understanding plant invasions.

## Supplementary Information

Supplementary Table S1.

## Data Availability

All sample data used in the analyses are available from figshare at: 10.6084/m9.figshare.18255068.
