# Supplementary material for: Road traffic and landscape characteristics predict the occurrence of native halophytes on roadside verges
Source: Sci Rep. 2022 Jan 25;12:1298. doi: 10.1038/s41598-022-05084-3 (PMC8789788; doi:10.1038/s41598-022-05084-3)
Supplement: Supplementary file 1 — Supplementary Table S1. [file 41598_2022_5084_MOESM1_ESM.pdf]

Table S1. – Other halophytes found at the sampling localities

Species	Number of localities, where found
Matricaria recutita	69
Atriplex tatarica	30
Plantago coronopus	19
Artemisia pontica	8
Spergularia rubra	7
Plantago tenuiflora	4
Lepidium perfoliatum	4
Myosurus minimus	4
Ranunculus pedatus	4
Plantago maritima	4
Artemisia santonicum	3
Hordeum hystrix	3
Spergularia salina	3
Pholiurus pannonicus	1
Sedum caespitosum	1
